# suMRak: a multi-tool solution for preclinical brain MRI data analysis

**DOI:** 10.3389/fninf.2024.1358917

**Published:** 2024-03-26

**Authors:** Rok Ister, Marko Sternak, Siniša Škokić, Srećko Gajović

**Affiliations:** ^1^Croatian Institute for Brain Research, University of Zagreb School of Medicine, Zagreb, Croatia; ^2^BIMIS—Biomedical Research Center Šalata, University of Zagreb School of Medicine, Zagreb, Croatia

**Keywords:** neuroimaging, magnetic resonance imaging, brain segmentation, image registration, MATLAB, Python

## Abstract

**Introduction:**

Magnetic resonance imaging (MRI) is invaluable for understanding brain disorders, but data complexity poses a challenge in experimental research. In this study, we introduce suMRak, a MATLAB application designed for efficient preclinical brain MRI analysis. SuMRak integrates brain segmentation, volumetry, image registration, and parameter map generation into a unified interface, thereby reducing the number of separate tools that researchers may require for straightforward data handling.

**Methods and implementation:**

All functionalities of suMRak are implemented using the MATLAB App Designer and the MATLAB-integrated Python engine. A total of six helper applications were developed alongside the main suMRak interface to allow for a cohesive and streamlined workflow. The brain segmentation strategy was validated by comparing suMRak against manual segmentation and ITK-SNAP, a popular open-source application for biomedical image segmentation.

**Results:**

When compared with the manual segmentation of coronal mouse brain slices, suMRak achieved a high Sørensen–Dice similarity coefficient (0.98 ± 0.01), approaching manual accuracy. Additionally, suMRak exhibited significant improvement (*p* = 0.03) when compared to ITK-SNAP, particularly for caudally located brain slices. Furthermore, suMRak was capable of effectively analyzing preclinical MRI data obtained in our own studies. Most notably, the results of brain perfusion map registration to T2-weighted images were shown, improving the topographic connection to anatomical areas and enabling further data analysis to better account for the inherent spatial distortions of echoplanar imaging.

**Discussion:**

SuMRak offers efficient MRI data processing of preclinical brain images, enabling researchers' consistency and precision. Notably, the accelerated brain segmentation, achieved through K-means clustering and morphological operations, significantly reduces processing time and allows for easier handling of larger datasets.

## Introduction

Magnetic resonance imaging (MRI) is a powerful tool for non-invasive imaging of the brain, and in recent years, it has significantly enhanced our understanding of brain anatomy, function, and pathology. In the field of preclinical research, MRI plays a pivotal role in studying animal models of various neurological disorders, investigating treatment efficacy, and understanding the underlying mechanisms of brain diseases (Denic et al., 2011; Kurniawan, 2018; Ramos-Cabrer and Padro, 2018). However, the large amount of data generated by these scans can make it challenging to analyze and extract meaningful information.

Researchers are often required to navigate through separate, unconnected applications, leading to inefficiencies, potential data inconsistencies, and increased analysis time. Moreover, the absence of unified tools limits the ability to perform complex analyses, hindering the depth of insights that can be gained from preclinical MRI data. Although there are many bioinformatics tools for MRI neuroimaging available, most of them are specialized for a narrow dataset type (e.g., human brain scans) or a specific use case (e.g., diffusion data analysis) (Man et al., 2015).

**Figure 1:**
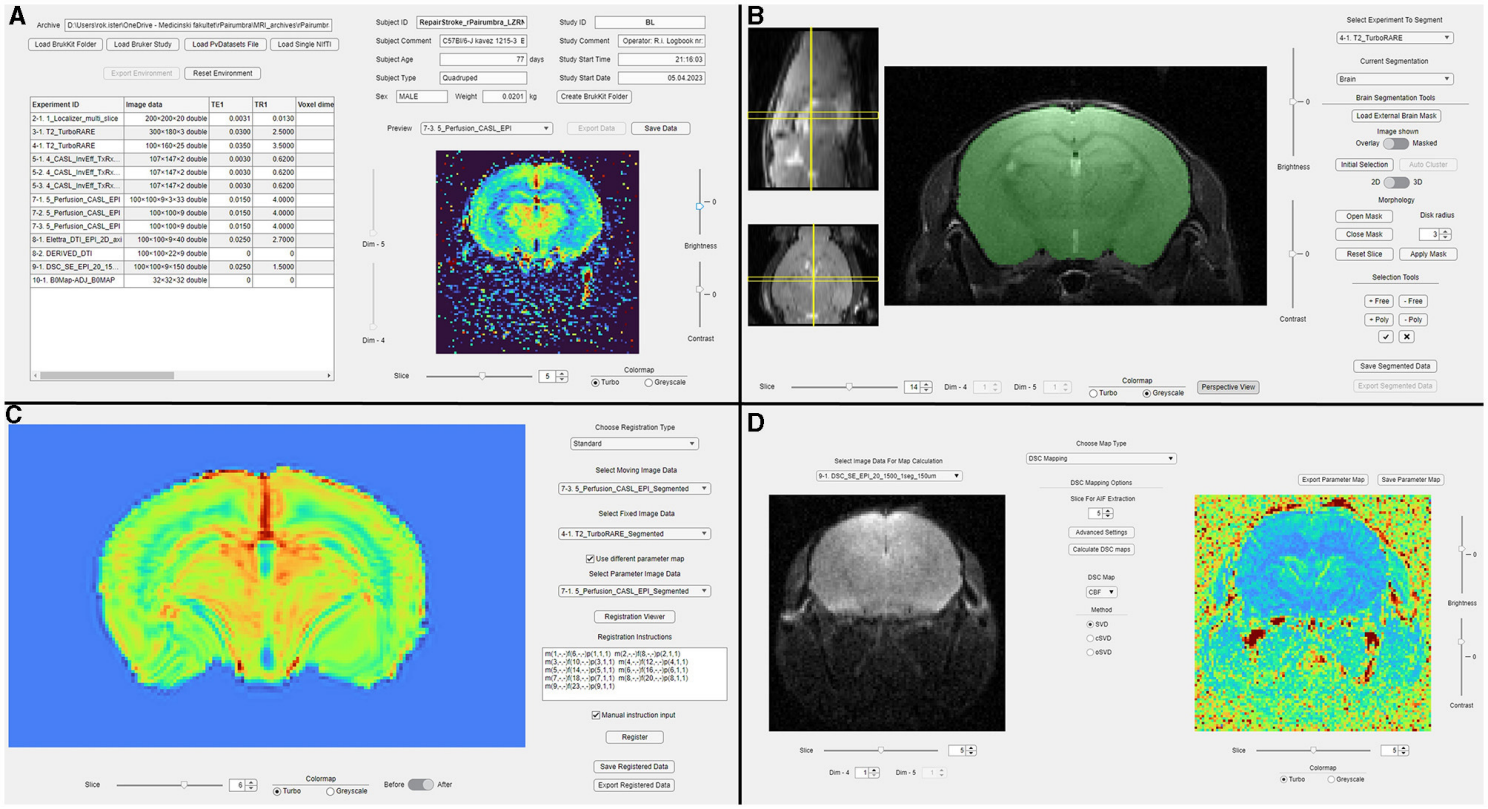
An overview of suMRak's graphical interfaces. **(A)** The main tab used for dataset importing and previewing imaging parameters, scan metadata, and reconstructed images. **(B)** The segmentation tab used for imported image data segmentation. **(C)** The registration tab used for registering a selected moving image to a fixed user-provided image or a reference atlas. **(D)** The parameter map tab used for the calculation of various parameter maps using imported image data.

In this study, we present the design and implementation of a novel MATLAB application, suMRak—a simple utility MRI analysis kit dedicated to brain imaging, encompassing essential modules: segmentation, volumetry statistic calculation, image data registration, and the generation of parameter maps (Figure 1). Additionally, suMRak offers a three-dimensional data viewer, thus enabling researchers to visualize brain structures in a user-friendly and intuitive manner. Finally, its functionality and effectiveness are demonstrated on real-world preclinical MRI datasets, showcasing its versatility and potential for advancing preclinical neuroimaging research.

## Methods and implementation

SuMRak was developed and compiled using the Graphical User Interface (GUI) building functionalities of MATLAB's App Designer under MATLAB releases R2021b and R2023a. As such, the application is available in the form of an App Designer binary *.mlapp* file and a fully exported MATLAB code *.m* file, both of which can be executed directly from the MATLAB Command Window, and as a single *.exe* installer file, which allows users to install suMRak (and the required MATLAB Runtime) as a standalone desktop application. Prerequisites for suMRak installation are Microsoft Visual C++ Redistributable 2015–2022 and a MATLAB R2023a-supported version of Python. Additionally, image registration requires a valid installation of the SimpleElastix Python library (Marstal et al., 2016), which can be compiled and installed by following the openly available SimpleElastix documentation. The application has been tested on 64-bit Windows 10 and Windows 11 but is compatible with 64-bit Windows 7 or later. SuMRak communicates and functions alongside a total of six helper applications, all of which are available as binary *.mlapp* and exported MATLAB code *.m* files. The helper apps and their functionalities are described within their relevant methodology sections below.

### Data importing and exporting

SuMRak accepts data in the form of singular Neuroimaging Informatics Technology Initiative (NIfTI) *.nii* files, Bruker ParaVision study folders, or *.PvDatasets* files, and custom-made suMRak folders.

Data from NIfTI files is loaded using the MATLAB Image Processing Toolbox functions *niftiread()* and *niftiinfo()* for image data and metadata, respectively.

Data from Bruker ParaVision study folders and *.PvDatasets* files is loaded using the proprietary Bruker *pvmatlab* package by initializing objects of the *ImageDataObject* class and scraping the image data and metadata. The *pvmatlab* package is available under its respective license from Bruker BioSpin MRI GmbH.

Custom suMRak folders containing exported work environment data as MATLAB *.mat* files are loaded using the MATLAB function *load()*, allowing users to continue a previously exported data analysis session where it was left off.

The following information is collected and stored from all loaded data types:

1. Experiment identification—the name of the experiment preceded by the experiment and processing number;
2. Experiment image data—loaded as a two-dimensional to five-dimensional double-precision floating-point matrix;
3. Experiment echo (TE) and repetition times (TR);
4. Voxel dimensions, slice thickness, and slice gap (distance between two adjacent slices), alongside respective units of measurement;
5. Experiment data rotation matrix used for proper exporting to NIfTI header metadata.

All data analyzed and generated using suMRak can be exported directly into NIfTI files.

### Segmentation

SuMRak provides straightforward brain, hemisphere, and region of interest (ROI) segmentation using a combination of *K*-means clustering, active contours, morphological operations, and manual polygonal or freehand ROI corrections.

#### Brain segmentation

*K*-means clustering is a popular unsupervised machine learning algorithm that aims to sort *n* data points into *k* different clusters by minimizing the distance between the data points within the same cluster. This minimization is done by iteratively recalculating an initially chosen central point (centroid) of each cluster to be the mean of all data points assigned to that cluster, that is until the centroids move less than a set threshold value or a pre-specified number of iterations is reached (Steinley, 2006; Arthur and Vassilvitskii, 2007). SuMRak implements two-dimensional and three-dimensional *K*-means clustering for brain segmentation using the MATLAB Image Processing Toolbox functions *imsegkmeans()* and *imsegkmeans3()*. After a rough initialization of the brain area, image data are partitioned into two clusters, one being the brain cluster itself and the other containing background image data. The correct cluster for further segmentation is selected based on a comparison between the Sørensen–Dice similarity coefficients of the respective cluster and the initial brain outline area, which is calculated using the following formula (Dice, 1945):


$$\begin{array}{l} SDSC=\frac{2\times |M_{cl}\cap M_{ba}|}{\left|M_{cl}|+|M_{ba}\;\right|}, \end{array}$$

where *M*_*cl*_ and *M*_*ba*_ represent binary masks of the analyzed cluster and the initial brain outline area, and |*M*_*cl*_| and |*M*_*ba*_| represent the number of elements in said binary masks. The coefficient value quantifies the similarity between binary masks by measuring the ratio of twice the number of their common elements to the total number of elements, providing a value between 0 (no similarity) and 1 (complete similarity). The brain cluster is selected as the one with the highest obtained coefficient.

Further refinement in the brain segmentation pipeline is available via morphological brain mask opening (morphological erosion followed by dilation) and closing (morphological dilation followed by erosion) using a variable-radius disk as a structuring element—implemented using the MATLAB Image Processing Toolbox functions *imopen()* and *imclose()*. Finally, any inconsistencies in the segmentation results can be corrected using manual freehand or polygonal ROI corrections.

#### Hemisphere segmentation

Hemisphere segmentation is available as a traditional freehand or polygonal selection of each hemisphere area, with the underlying prerequisite that a valid brain mask be used as the intersection reference—each pixel that is an element of a hemisphere mask must also be an element of the underlying brain mask. Furthermore, the brain mask is also used for optional hemisphere auto-completion. If the auto-completion option is selected, suMRak automatically adjusts the contralateral hemisphere mask to contain all the pixels that are elements of the brain mask but not of the currently segmented hemisphere.

#### Region of interest segmentation

Similarly, ROI segmentation is also based on a freehand or polygonal selection of the desired ROI area, with additional options of region growth using active contours (iterative curve adjustment for object edge outlining —useful for automatic blood vessel segmentation) (Kass et al., 1988) and automatic partitioning of the entire image volume into a predefined number of “superpixels”. These additional options are implemented as part of the ROIVolumeSegmenter helper application using the MATLAB Image Processing Toolbox functions activecontour() and superpixels3().

### Volumetric data calculation

Following image segmentation, summary statistics can be calculated for all resulting volume masks: the segmented brain, hemispheres, and all regions of interest. The following data are calculated automatically: mask total volume, mean voxel intensity, standard deviation, median, interquartile range, and minimum/maximum voxel intensity values. The total mask volume is calculated as a product of the number of voxels contained inside a given mask and a single slice voxel volume (V_vox_), which is obtained from the imported data as follows:


$$\begin{array}{l} V_{vox}=X_{vox}\times Y_{vox}\times Slice\;thickness, \end{array}$$

where X_vox_ and Y_vox_ represent the respective voxel dimensions. To account for the volume within slice gaps that are missed using this approach, a slice gap correction volume (CV_gap_) is added to the total mask volume, which is calculated as the sum of all single gap volumes for slice gaps adjacent to non-zero mask slices on both sides:


$$\begin{array}{l} CV_{gap}\overset{n}{\underset{i=1}{\sum }}A_{gap_{i}}\times Slice\;gap, \end{array}$$

where A_gap_ represents the area of a given slice gap, estimated as the average of the two adjacent mask slice areas (A_back_, A_front_), which can be easily derived from their respective number of voxels (N_back_, N_front_) and voxel dimensions (X_vox_, Y_vox_):


$$\begin{array}{l} A_{gap}=\frac{A_{back}+A_{front}}{2}=\;\frac{N_{back}+N_{front}}{2}\times X_{vox}\times Y_{vox}\; \end{array}$$

### Edema correction of the region of interest

For imaging experiments studying disease models in which post-trauma brain swelling may occur (stroke, traumatic brain injury, etc.), all ROI mask volumes can be adjusted for brain edema using one of three available correction methods, assuming valid hemisphere segmentation. The first method of correction uses a hemisphere scaling factor described by Reglodi et al. (2002), where the corrected ROI area for each slice is obtained as a product of the original ROI area and a ratio of the contralateral and ipsilateral hemisphere areas:


$$\begin{array}{l} A_{corrected}=\;A_{original}\times \frac{A_{contralateral}}{A_{ipsilateral}} \end{array}$$

The second method uses a correction factor described by Belayev et al. (2003), which is calculated as the complement of a ratio of hemisphere area difference and contralateral hemisphere area:


$$\begin{array}{l} A_{corrected}=\;A_{original}\times \left(1-\frac{{A_{ipsilateral}-A}_{contralateral}}{A_{contralateral}}\right)\; \end{array}$$

The final method, a version of the one described by Gerriets et al. (2004), modified for areas instead of volumes, calculates the corrected ROI area for each slice from hemisphere areas as follows:


$$\begin{array}{l} A_{corrected}=A_{contralateral}+\;A_{ipsilateral}\\ \;-\left(A_{contralateral}+\;A_{ipsilateral}-A_{original}\right)\\ \;\times \frac{A_{contralateral}+\;A_{ipsilateral}}{2\times A_{contralateral}} \end{array}$$

All obtained corrected ROI areas—using any of the aforementioned correction methods—are then multiplied by the slice thickness to obtain the final corrected ROI volume. Finally, the slice gap correction volume CV_gap_ calculation is then modified to use edema-corrected slice areas for slice gap area estimation.

### Registration

SuMRak utilizes the MATLAB-integrated Python engine to enable imported image data registration by passing instructions to the SimpleElastix Python library (Marstal et al., 2016) using the MATLAB function pyrun(). Three main forms of registration are implemented: standard non-rigid moving/fixed registration, reference atlas registration, and time-series data alignment.

In standard moving/fixed registration, the default SimpleElastix non-rigid parameter map vector (using the affine and B-spline transformation models) is used to align images by incorporating localized deformations—accommodating anatomical, physiological, and pathological variations that cannot be achieved by rigid registration alone. When registering an MRI parameter map, more precise registration can be achieved by using a third experiment (“parameter image”), which is registered to the fixed image data first, and the optimized transformation parameter map is retrieved for the subsequent registration of the original moving image to the fixed image data (e.g., a parameter map—moving image, and its raw, pre-processed data—parameter image).

Reference atlas registration is enabled with the same workflow capabilities as standard moving/fixed registration, with the target fixed image data being a reference MRI or histological atlas, instead of an imported imaging experiment. All reference atlas data are downloaded, imported, and optionally saved for future usage through the ReferenceAtlasImporter helper application. Currently, supported anatomical reference atlases include the Allen Brain Atlas adult mouse Nissl grayscale atlas (Allen Instutiute for Brain Science, 2004), T1 and T2 weighted Waxholm Space Atlases of the C57Bl/6j mouse (NITRC, 2023), and the Mouse Imaging Centre Neuroanatomy Atlas of the C57Bl/6j mouse (Dorr et al., 2008).

Prior to either standard moving/fixed or reference atlas registration, the suMRak helper applications RegistrationViewer_Basic and RegistrationViewer_Parameter enable easier instruction generation by providing side-by-side moving, fixed, and parameter image data viewing options.

Time-series data alignment is enabled for four-dimensional and five-dimensional data, which is intended for spatiotemporal realignment of functional MRI (fMRI) sequences. The registration process uses a default SimpleElastix rigid transformation model under the assumption that the motion-related artifacts that occur during image acquisition in these sequences are related to a chosen reference data point only by rotation and translation. After a reference fourth (and in the case of five-dimensional data, a fifth) dimension data point is selected by the user; all other data points are registered to the reference point using the parallel processing capabilities of the parfor loop in the MATLAB Parallel Computing Toolbox.

### Parameter map calculation

A variety of different parameter maps can be calculated in suMRak from original or pre-processed data: T1, T2, pulsed arterial spin labeling (pASL), and dynamic susceptibility contrast (DSC) maps.

#### T1 and T2 maps

T1 and T2 mapping in MRI are quantitative techniques that provide numerical values, reflecting the inherent properties of imaged tissues. T1 mapping involves acquiring images with varying repetition or inversion times to measure the longitudinal or spin-lattice relaxation time (T1), while T2 mapping employs images with different echo times to assess the transverse or spin-spin relaxation time (T2).

T1 and T2 map calculation in suMRak uses an iterative least-squares curve fitting approach by applying the Levenberg–Marquardt optimization algorithm to fit an exponential decay model to normalized MRI data (Levenberg, 1944; Marquardt, 1963), effectively calculating T1 and T2 relaxation times for each individual voxel. This calculation is implemented using the MATLAB Optimization Toolbox function lsqcurvefit().

#### Pulsed arterial spin labeling maps

Pulsed arterial spin-labeled map calculation involves acquiring pairs of MRI images with and without magnetically labeled arterial blood, subtracting them to obtain the perfusion-weighted signal, and then converting this signal into a cerebral blood flow (CBF) map.

SuMRak calculates relative perfusion maps from raw pASL data using a similar approach to T1 map calculation, fitting a two-parameter model to normalized T1 values for both labeled and non-labeled conditions using the Levenberg–Marquardt optimization algorithm (Levenberg, 1944; Marquardt, 1963). The resulting relative perfusion values are then computed based on the T1 value of blood.

#### Dynamic susceptibility contrast maps

Dynamic susceptibility contrast (DSC) imaging begins with an intravenous injection of a gadolinium bolus, followed by a rapid acquisition of T2-weighted images to track the passage of the contrast agent through the brain's vasculature. By analyzing the signal intensity-time curve derived from dynamic images, DSC imaging provides valuable information about cerebral blood flow (CBF), cerebral blood volume (CBV), and mean transit time (MTT), aiding in the assessment of brain perfusion.

Given valid DSC imaging data, suMRak calculates CBF, CBV, and MTT maps using the DSC MRI Toolbox for MATLAB authored by Peruzzo D. and Castellaro M., making use of the available arterial input function extraction tool (Peruzzo et al., 2011) and deconvolution algorithms (Zanderigo et al., 2009; Peruzzo et al., 2017).

### 3D Viewer

The functionalities of the three-dimensional data viewer are implemented using the MATLAB Image Processing Toolbox function volshow() and the parent Viewer3D object. This enables suMRak to offer a multitude of different data rendering styles, including volume rendering, minimum/maximum intensity projections, slice planes, isosurface rendering, and gradient opacity. Furthermore, nine MATLAB colormaps and three preset alphamaps are available to choose from, with optional interactive alphamap adjustment. Finally, the OverlayPicker helper application allows users to easily overlay imported or already processed data onto the one currently viewed.

### Test data

Alongside the main application files, suMRak includes two Bruker Paravision study folders and two .PvDatasets files used for application testing. These contain 38 different *in vivo* datasets obtained on adult C57BL/6J mice and eight *ex vivo* imaging experiments using a banana as the object under investigation. All figures shown contain data from these test files and were processed using suMRak.

### Segmentation validation

To validate our segmentation strategy, suMRak was compared against ITK-SNAP version 4.0.2 (Yushkevich et al., 2006) and fully manual segmentation in Fiji (Schindelin et al., 2012), which was set as the ground truth. ITK-SNAP is a popular open-source software application specialized for biomedical image segmentation, offering a semi-automatic segmentation pipeline using active contours. A total of 10 coronal mouse brain slices from two different T2-weighted imaging experiments were selected using a random number generator and were then segmented manually by two trained independent researchers. The resulting brain masks were subsequently compared with those obtained using only semi-automatic segmentation methods—namely *K*-means clustering followed by mask opening and closing for suMRak and active contours with thresholding pre-segmentation for ITK-SNAP. This comparison was done by calculating Sørensen–Dice similarity coefficients and their average value for every pair of comparisons to manual segmentation masks of the same brain slice. Finally, the obtained average coefficients for suMRak and ITK-SNAP were compared using a paired samples *t*-test, with the statistical significance level set at α = 0.05.

All data from these comparisons are reported in text as the arithmetic mean ± standard deviation.

## Results

### Segmentation

**Figure 2:**
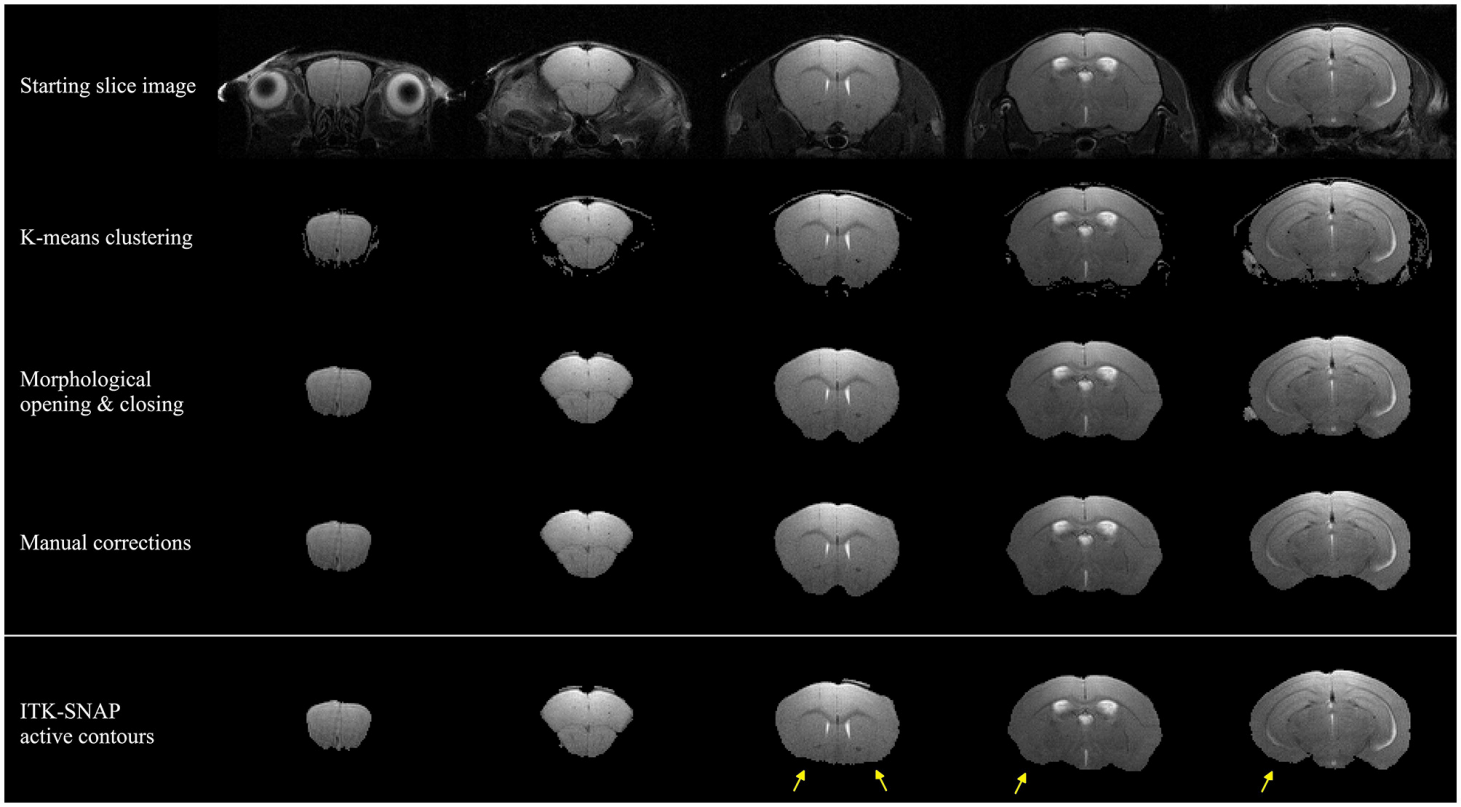
Segmentation results of five coronal mouse brain slices imaged with a T2-weighted imaging sequence. The upper three rows depict suMRak standard segmentation steps: *k*-means clustering, morphological opening and closing, and manual corrections. The bottom row depicts ITK-SNAP segmentation results in which the active contour method fails to extend to anterior brain regions (yellow arrows).

The SuMRak segmentation pipeline was applied to five different coronal brain slices in a T2-weighted imaging sequence (Figure 2). All slices were segmented using the same workflow order, starting with initial brain region K-means clustering, followed by morphological brain mask opening and closing, and finishing with manual corrections using traditional polygonal ROI selection.

Most of the segmentation processes in suMRak are condensed in the automated clustering and morphological operations, with most slices requiring little to no manual corrections. This finding was further confirmed in the validation and comparison process of suMRak to manual segmentation, which showed that without manual corrections, suMRak clustering and morphological operations achieved an average Sørensen–Dice similarity coefficient of 0.98 ± 0.01. Furthermore, the semi-automatic segmentation offered by ITK-SNAP achieved an average Sørensen–Dice similarity coefficient of 0.94 ± 0.06, and when compared with the results from suMRak using a paired samples *t*-test, the difference was found to be statistically significant (*p* = 0.03). This difference is most notable in the caudal region of the mouse brain (Table 1, slices 23 and 21), where suMRak performs much better than active contours in ITK-SNAP.

**Table 1:** Resulting Sørensen–Dice similarity coefficients of suMRak and ITK-SNAP when compared with manual segmentation of 10 different coronal mouse brain slices in Fiji.

**Mouse ID**	**Slice number**	**suMRak/manual segmentation**	**ITK-SNAP/manual segmentation**
1550	2	0.98	0.96
	3	0.98	0.97
	11	0.98	0.95
	15	0.99	0.98
	23	0.97	0.82
1582	3	0.98	0.94
	7	0.98	0.96
	14	0.99	0.97
	16	0.99	0.97
	21	0.95	0.85

Each coefficient represents an average value of the coefficient pairs obtained when comparing suMRak/ITK-SNAP slice masks with the two different manually segmented masks for the corresponding brain slice. Caudal slices nos. 21 and 23 exhibited the greatest observed differences (highlighted in green).

### Registration

**Figure 3:**
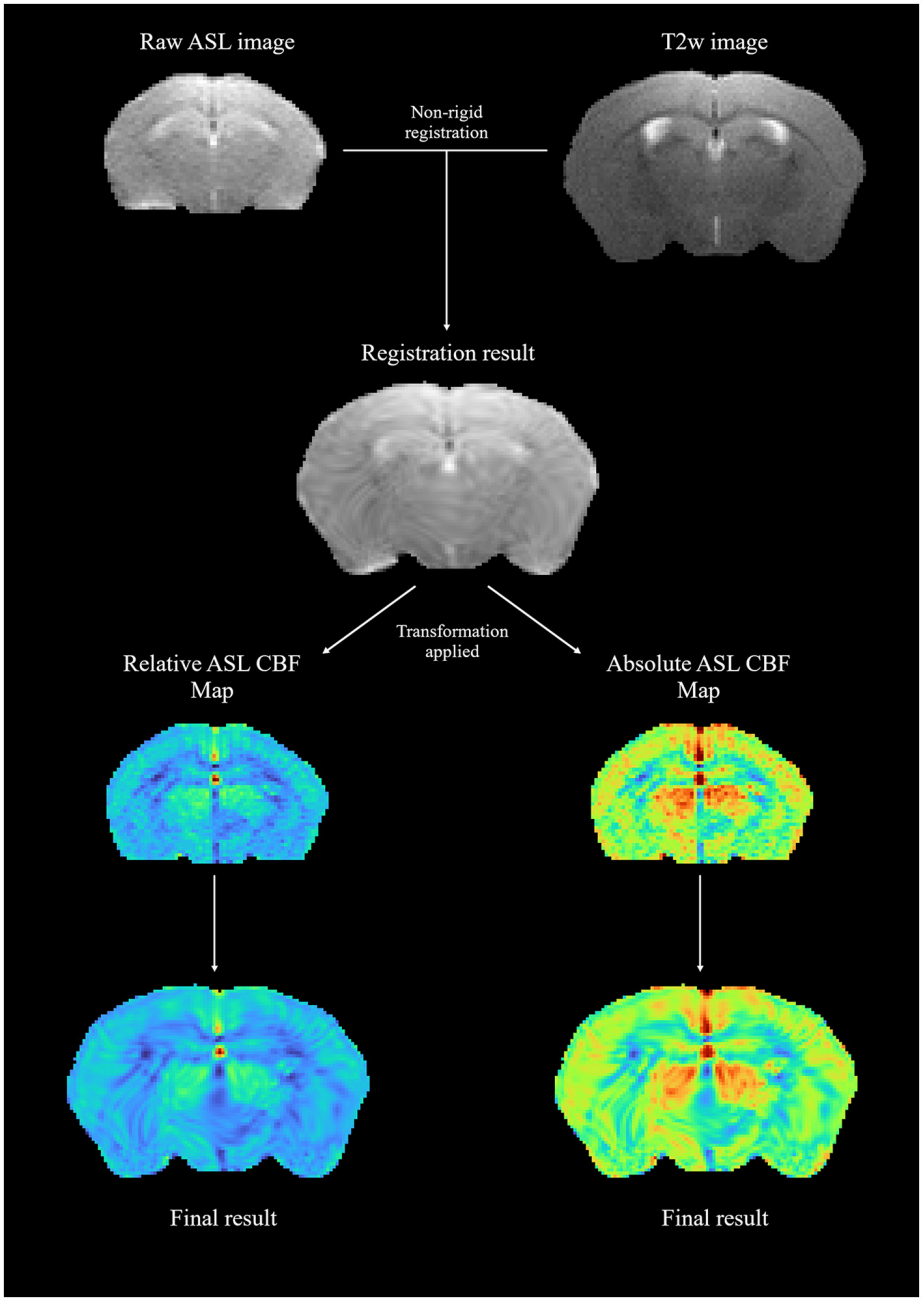
A sample moving/fixed registration workflow; raw ASL image data were registered onto a T2-weighted image to obtain a transformation parameter map, which was then applied to relative and absolute ASL-CBF maps.

Figure 3 shows a sample standard moving/fixed registration workflow in which central brain slices of arterial spin labeling cerebral blood flow (ASL-CBF) maps were registered onto a corresponding T2-weighted image to account for spatial distortions inherent in echoplanar imaging (EPI) acquisition and, in turn, improve the topographical connection to anatomical areas. By using the raw ASL image data and registering it to the T2-weighted image first, a more precise transformation parameter map was obtained for map registration, which was subsequently applied to both relative and absolute ASL-CBF maps to obtain the final registration results.

### Parameter maps

**Figure 4:**
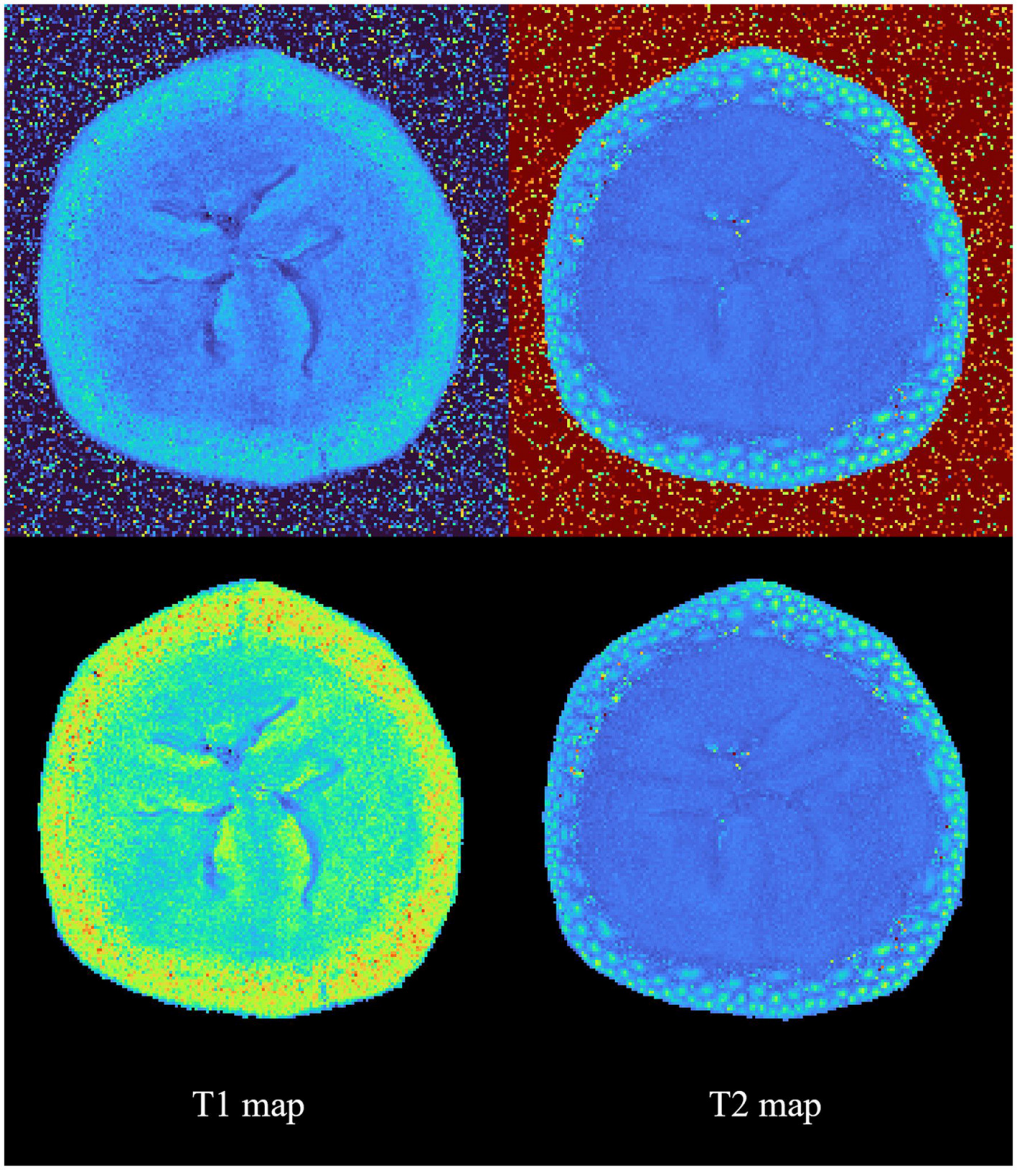
Sample calculation results of T1 and T2 parameter maps with and without prior segmentation. This figure shows suMRak's capability to handle a wide variety of MRI data.

Sample T1 and T2 parameter maps were generated with and without prior segmentation of a banana imaging experiment and are shown in Figure 4. By incorporating segmentation prior to map generation within a unified interface, suMRak effectively eliminated background noise, showcasing its versatility in this relatively unusual imaging experiment.

### 3D viewer

**Figure 5:**
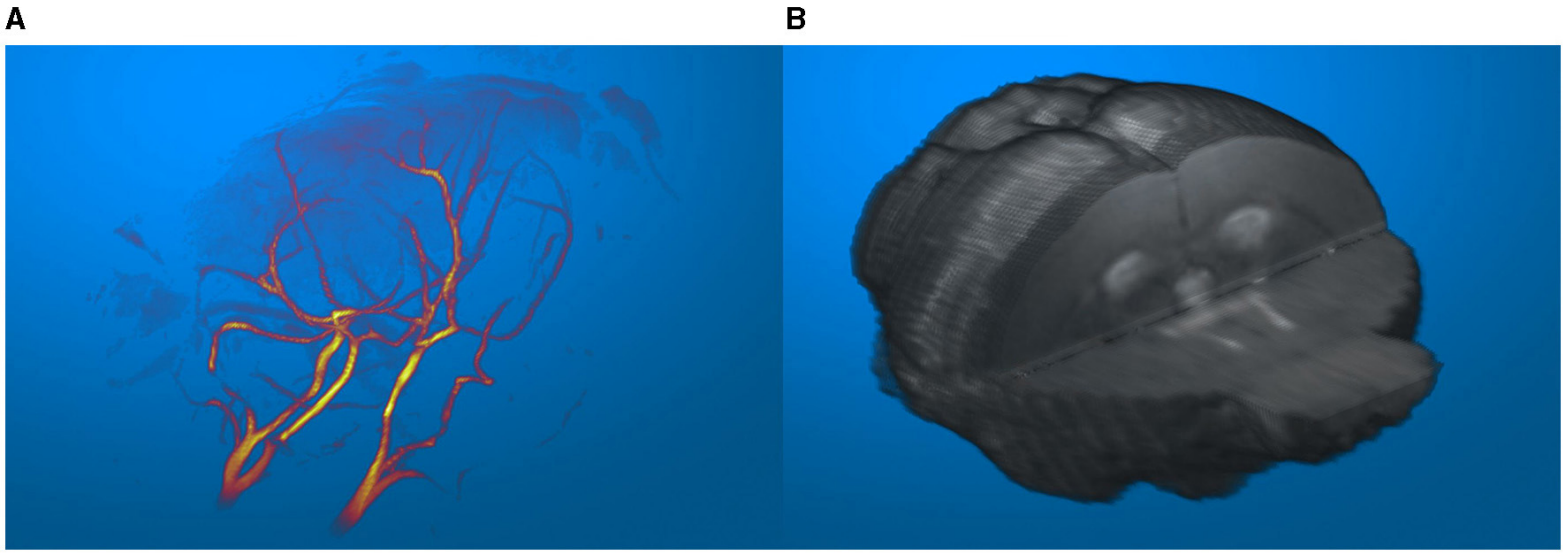
**(A)** Blood vessels of an adult mouse brain, visualized in an imported MR angiography sequence. **(B)** Segmented mouse brain from a T2-weighted image visualized in 3D.

The three-dimensional data visualization capabilities of suMRak are presented in Figure 5, which shows different options for rendering three-dimensional volume from multislice 2D MRI data. The blood vessels of an adult mouse brain in Figure 5A were visualized from a gradient echo angiography sequence data using a maximum intensity projection option and displayed applying the MATLAB colormap hot. Figure 5B shows a three-dimensional visualization of the same mouse brain segmented from a T2-weighted image using the volume rendering method.

## Discussion

In this study, we presented an overview of the functionalities provided by the novel MATLAB application for preclinical brain MRI analysis—suMRak along with the sample results obtained by testing it on accompanying real-world datasets.

The integration of brain segmentation, volumetric statistics calculation, image data registration, parameter map generation, and a 3D data viewer into a unified interface marks a fundamental aspect of suMRak. A seamless transition between various analyses without the need for multiple standalone tools fosters a cohesive and streamlined workflow, which not only saves time but also ensures consistency across different analyses—minimizing errors and increasing the reliability of results. For instance, by using suMRak, a researcher can import raw image data, segment a region of interest, calculate its parameter map, register it to a reference anatomical scan, extract the ROI statistics, and, optionally, export it to a NifTI file with the correct header set, all in a singular environment. To the best of our knowledge, none of the available MRI analysis tools are able to provide that functionality.

The enhanced speed of brain segmentation achieved by our application stands out among its core utilities. By leveraging *K*-means clustering and morphological operations, suMRak greatly reduces the processing time for brain structures, thus enabling more efficient work with larger datasets. This finding was confirmed by comparing suMRak with manual segmentation of coronal mouse brain slices, with the obtained data showing that suMRak achieved an average Sørensen–Dice similarity coefficient of 0.98 ± 0.01—a result that demonstrates how close *K*-means clustering and morphological operations get to the accuracy of fully manual segmentation. Furthermore, when compared with semi-automatic segmentation offered by ITK-SNAP through active contours (average Sørensen–Dice similarity coefficient of 0.94 ± 0.06), suMRak showed significant improvement, especially for brain slices located caudally. Moreover, it is important to mention that, although suMRak also provides active contour segmentation for regions of interest, this method may not be appropriate for rostral mouse brain slices. This limitation arises because the brain mask can readily extend to the eyes and optic tract due to similar voxel intensities on a T2-weighted image. Finally, all segmentation masks generated in suMRak are easily exportable and reusable, allowing for greater consistency across work sessions and transparent error-checking.

While suMRak currently accepts a relatively narrow range of file formats for data importing, the standardized set of information was extracted from all data types and stored in a singular MATLAB table format, allowing for straightforward implementation of support for other data types in the future.

In conclusion, suMRak represents an advancement in the realm of preclinical brain MRI analysis. By addressing the need for integration and speed, suMRak stands out as a versatile tool that can help researchers process their MRI data more efficiently and redirect saved time into other fields of interest.

## Data availability statement

The datasets presented in this study can be found in online repositories. The names of the repository/repositories and accession number(s) can be found below: https://github.com/hiim-hr/suMRak/releases.

## Ethics statement

The animal study was approved by Ethical Committee of University of Zagreb School of Medicine. The study was conducted in accordance with the local legislation and institutional requirements.

## Author contributions

RI: Conceptualization, Data curation, Formal analysis, Investigation, Methodology, Software, Validation, Writing – original draft, Writing – review & editing. MS: Data curation, Formal analysis, Methodology, Software, Validation, Visualization, Writing – original draft, Writing – review & editing. SŠ: Investigation, Methodology, Software, Supervision, Validation, Writing – review & editing. SG: Funding acquisition, Project administration, Resources, Supervision, Writing – review & editing.
